# Novel *AGL* variants in a patient with glycogen storage disease type IIIb and pulmonary hypertension caused by pulmonary veno-occlusive disease: A case report

**DOI:** 10.3389/fgene.2023.1148067

**Published:** 2023-03-23

**Authors:** Akito Shindo, Kazutaka Ueda, Shun Minatsuki, Yukiteru Nakayama, Satoshi Hatsuse, Kanna Fujita, Seitaro Nomura, Masaru Hatano, Norifumi Takeda, Hiroshi Akazawa, Issei Komuro

**Affiliations:** Department of Cardiovascular Medicine, Graduate School of Medicine, The University of Tokyo, Tokyo, Japan

**Keywords:** pulmonary hypertension, pulmonary veno-occlusive disease, PVOD, glycogen storage disease, AGL

## Abstract

Glycogen storage disease type III (GSD-III) is an autosomal recessive metabolic disorder caused by mutations in the *AGL* gene, and may develop various types of pulmonary hypertension (PH). Here, we report a case of 24-year-old man with GSD-IIIb with two novel null variants in *AGL* (c.2308 + 2T>C and c.3045_3048dupTACC). He developed multi-drug-resistant pulmonary veno-occlusive disease (PVOD) and was registered as a candidate for lung transplantation. No pathogenic variants were detected in previously known causative genes for pulmonary hypertension and the underlying mechanism of coincidence of two rare diseases was unknown. We discuss the association of the loss of glycogen-debranching enzyme with incident PVOD.

## Introduction

Glycogen storage disease type III (GSD-III; OMIM 232400, ORPHA 366) is a rare autosomal recessive hepatic disease in which glycogen abnormally accumulates in the liver. This inherited disorder is rare, with an estimated prevalence of 1:100,000 (Sentner et al., 2016). The mutation in the *AGL* gene, which encodes a glycogen debranching enzyme, is known to cause GSD-III. Glycogen debranching enzymes degrade phosphorylase-limited dextrin, a form of glycogen broken down by phosphorylase α, to glucose 1-phosphate (Sentner et al., 2016). Enzymatic dysfunction of glycogen debranching enzymes results in tissue deposition of phosphorylase-limited dextrin. In total, 85% of GSD cases are type IIIa, which affect the skeletal and cardiac muscles in addition to the liver, while 15% are type IIIb, which only affect the liver and has no effect on muscles. Types IIIc and d also exist but are considered rare conditions. Patients with GSD are known to be susceptible to group 5 pulmonary hypertension (PH), PH due to unclear multifactorial mechanisms (Simonneau et al., 2013). To date, most reports describing cases of GSD complicated by PH have been type I or II (Pizzo, 1980; Hamaoka et al., 1990; Ohura et al., 1995; BolzF Stocker and Zimmermann, 1996; Kishnani et al., 1996; Noori et al., 2002; Ueno et al., 2009; Kobayashi et al., 2010; Banka et al., 2011; Banka and Newman, 2013; Tavil et al., 2014; Yang et al., 2015; Li et al., 2018; Kim et al., 2020), while only a few reports have described GSD-III combined with PH (Lee et al., 2011) (Table 1).

**TABLE 1:** Glycogen storage disease and pulmonary hypertension: review of the literature. GSD, glycogen storage disease; ERT, enzyme replacement therapy; PVOD, pulmonary veno-occulusive disease; PAH, pulmonary arterial hypertension.

year	Pt. included	Pt. with PH	Age	GSD type	Therapy	Death	Lung pathology	Reference
**2020**	44	2	22,27	Ia	medication	-	-	Kim et al. (2020)
**2018**	1	1	16	II	medication, supportive	-	-	Li et al. (2018)
**2015**	1	1	16	II	ERT	-	-	Yang et al. (2015)
**2014**	1	1	0	Ia	medication	-	-	Tavil et al. (2014)
**2013**	57	5	0,0,2,13,38	Ia	-	-	-	Banka and Newman (2013)
**2011**	2	2	13,16	III	medication	+	-	Lee et al. (2011)
**2011**	4	1	0	Ia	medication	-	-	Banka et al. (2011)
**2010**	4	1	29	II	ERT	+	PVOD	Kobayashi et al. (2010)
**2009**	1	1	17	Ia	medication	-	-	Ueno et al. (2009)
**2002**	1	1	0	II	-	-	-	Noori et al. (2002)
**1996**	1	1	4	I	-	+	PAH-like	BolzF Stocker and Zimmermann (1996)
**1996**	1	1	24	Ia	medication	-	-	Kishnani et al. (1996)
**1995**	1	1	21	Ia	-	+	-	Ohura et al. (1995)
**1990**	2	2	12,16	I	-	+	PAH-like	Hamaoka et al. (1990)
**1980**	1	1	16	I	-	+	PAH-like	Pizzo (1980)

Pulmonary veno-occlusive disease (PVOD) is commonly one of the causes of group 1 PH and accounts for 5%–25% of all PH cases, with a prevalence of 1-2 per million people (Montani et al., 2016). The prognosis of PVOD is poor, with studies reporting a 1-year mortality of up to 72% (Holcomb et al., 2000), and lung transplantation is the only curative therapy.

There are very few cases of PVOD associated with GSD, with only one report of PVOD in GSD type I and no report of PVOD in GSD-III (Kobayashi et al., 2010). In this report, we described a rare case of GSD-IIIb with PVOD-induced PH. Whole-exome sequencing of this case identified two novel null variants in *AGL,* the causative gene for GSD-III, presumably present in the compound heterozygous state. We present the clinical course of this case and discuss the possibility that the newly discovered *AGL* mutations provoke PVOD. GSD-IIIb is considered to have good prognosis but can be complicated by severe PH requiring careful follow-up.

## Case presentation

**FIGURE 1:**
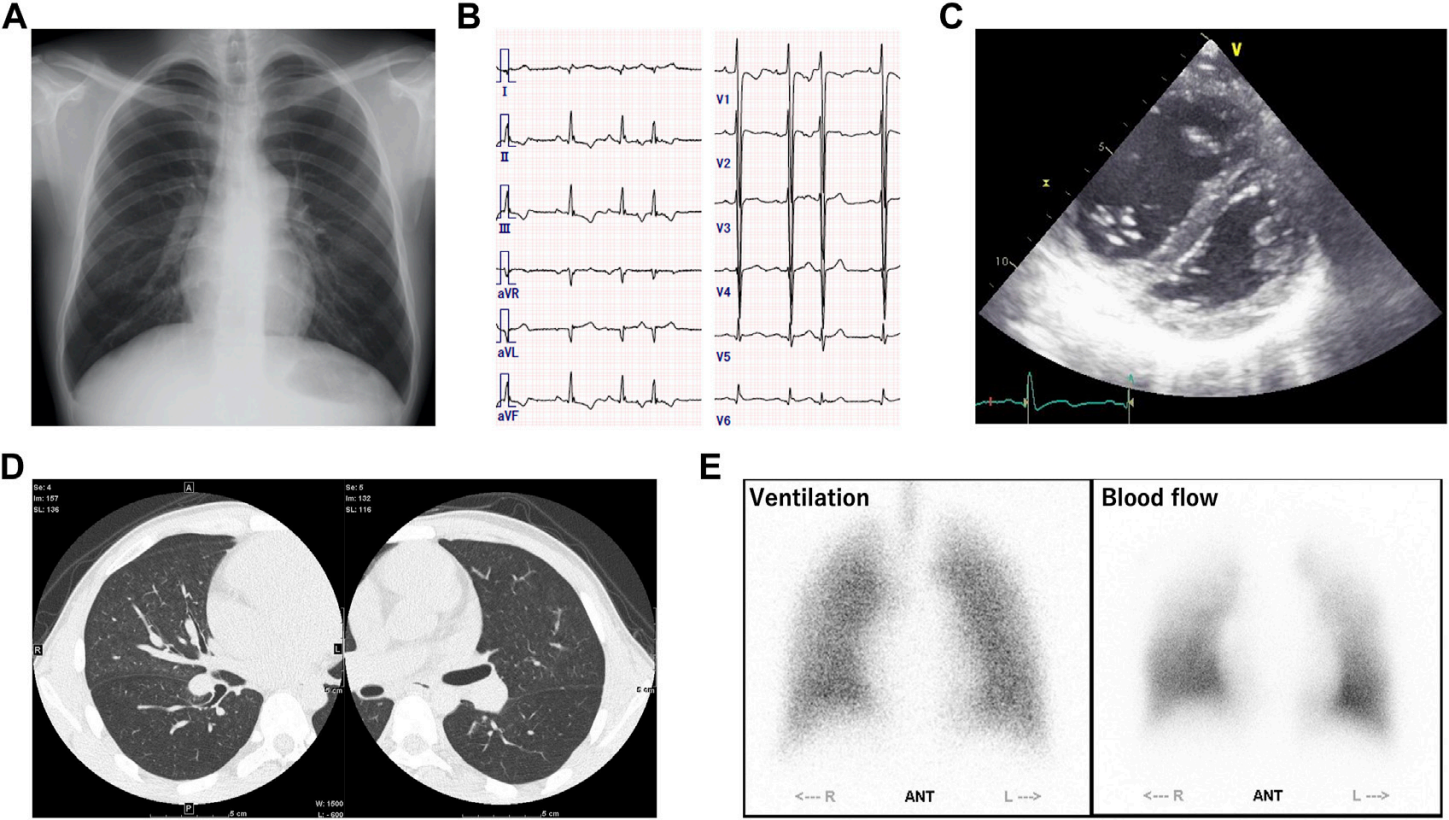
**(A)** Chest radiography revealed dilatation of both pulmonary arteries. **(B)** Electrocardiography showed right-axis deviation, increased V1 R wave, and negative T waves in II/III/aVF/V2, indicating right ventricular strain. **(C)** Echocardiography revealed right ventricular dilation and flattening of the interventricular septum, and the estimated right ventricular systolic pressure was elevated to 64 mmHg. **(D)** Contrast-enhanced computed tomography of the chest showed centrilobular granular shadows and ground glass opacities in both lungs, and no thrombus was present in the pulmonary arteries. **(E)** Pulmonary ventilation scintigraphy showed no deficit, and pulmonary blood flow scintigraphy showed mild physiological hypointensity in the upper lobes of both lungs.

A 24-year-old male was admitted to our hospital for close examination of shortness of breath on exertion. He was clinically diagnosed with GSD-III due to hepatomegaly and elevated liver enzyme levels at 6 months old; however, his subsequent growth and development were asymptomatic. Since he has no muscular symptoms nor cardiac symptoms at that time, his GSD was considered to be type Ⅲb. The patient had no family history of patients with GSD or PH. At 22 years of age, he became aware of the shortness of breath on exertion, which gradually worsened. Hypoxemia was observed at rest with SpO_2_ 86% and pO_2_ 58.8 mmHg on the arterial blood gas test, and the intensity of the pulmonic component of the second heart sound was enhanced. Blood tests revealed mildly elevated levels of transaminases (aspartate aminotransferase (AST), 82 IU/L; alanine aminotransferase (ALT), 96 IU/L) and brain natriuretic peptide (BNP, 81 pg/mL). Although mild elevations in antinuclear and anti–Sjögren syndrome-related antigen A (anti-SSA) antibody levels were observed, autoimmune diseases were carefully excluded through physical examination by rheumatologists. Chest radiography revealed dilatation of both pulmonary arteries (Figure 1A), and electrocardiography showed right-axis deviation, increased V1 R wave, and negative T waves in II/III/aVF/V2, indicating right ventricular strain (Figure 1B). Echocardiography revealed right ventricular dilation and flattening of the interventricular septum, and the estimated right ventricular systolic pressure was elevated to 64 mmHg (Figure 1C). Right heart catheterization revealed PH with a mean pulmonary artery pressure (mPAP) of 53 mmHg, mean pulmonary wedge pressure (mPCWP) of 15 mmHg, and pulmonary vascular resistance (PVR) of 10.7 Wood units. Sampling of the oxygen content at multiple sites and echocardiographic bubble study ruled out systemic-pulmonary shunt. The 6-min walk test distance was 550 m. Contrast-enhanced computed tomography of the chest showed centrilobular granular shadows and ground glass opacities in both lungs, and no thrombus was present in the pulmonary arteries (Figure 1D). Pulmonary ventilation scintigraphy showed no deficit, and pulmonary blood flow scintigraphy showed mild physiological hypointensity in the upper lobes of both lungs (Figure 1E). Respiratory function tests showed a marked decrease in pulmonary diffusion capacity with a vital capacity percentage (%VC) of 105.7%, forced vital capacity (FVC) of 4.87 L, forced expiratory volume in one second (FEV_1_) of 3.66 L, diffusing capacity for carbon monoxide (DLCO) of 8.62 mL/min/mmHg, and %DLCO of 32.5%. Based on these results, the patient was clinically diagnosed with PVOD and treated with riociguat (7.5 mg), macitentan (10 mg), and home-based oxygen therapy.

Whole-exome sequencing was performed on this case and revealed two likely pathogenic null variants, c.2308 + 2T>C and c.3045_3048dupTACC (p.Thr1017TyrfsX54), at the locus of *AGL*, a known causative gene for GSD-III (see Method and Supplementary Figure S1). In contrast, no pathological mutations were found in the *EIF2AK4* gene, a known causative gene for PVOD (Montani et al., 2016), or in any other PH-related genes. The patient’s shortness of breath on exertion did not improve after the start of medical treatment. Two months after the start of medical therapy, his 6-min walk test distance worsened to 401 m, and right heart catheterization revealed an mPAP of 50 mmHg and PVR of 7.9 Wood units, indicating a lack of response to medical therapy. Treatment courses other than lung transplantation were considered less effective, and the patient was registered as a candidate for lung transplantation. The average waiting period for lung transplantation in Japan is 2.5 years, and the patient is still receiving outpatient care after registration.

## Discussion

To the best of our knowledge, this is the first case report of GSD-IIIb complicated by PH due to PVOD. There have been several reports describing cases of GSD complicated by PH, but most of them were reports of GSD type I or II, and only two cases of GSD-III accompanied by PH have been reported (Table 1) (Pizzo, 1980; Hamaoka et al., 1990; Ohura et al., 1995; BolzF Stocker and Zimmermann, 1996; Kishnani et al., 1996; Noori et al., 2002; Ueno et al., 2009; Kobayashi et al., 2010; Banka et al., 2011; Lee et al., 2011; Banka and Newman, 2013; Tavil et al., 2014; Yang et al., 2015; Li et al., 2018; Kim et al., 2020). In the previous reports of GSD-III accompanied by PH, pulmonary arterial hypertension was diagnosed clinically.

In this case, whole-exome sequencing identified two previously unreported null variants in the *AGL* gene, c.2308 + 2T>C and c.3045_3048dupTACC. No pathological variants were found in the known genes associated with the development of PH. This result gives rise to the possibility that these *AGL* gene mutations are common cause of the pathogenesis of GSD-IIIb and PVOD. c.2308 + 2T>C variant disrupts the conserved GT donor splice site of intron 17 with a change of Shapiro–Senapathy (S&S) splice scores from 74.25 to 57.09 and is predicted to cause exon 17 skipping (151 bp) and c.3045_3048dupTACC is an out-of-frame variant in exon 23 (p.THr1017TyrfsX54), and thus these two mRNAs are presumably degradated by non-sense-mediated mRNA decay, leading to loss of all enzymatic activity of AGL and contributing to the onset and progression of GSD- IIIb and PVOD.

Since the chance of coincidental occurrence of the two rare diseases, GSD and PVOD, is extremely small, it seems reasonable to assume a connection between them. Although the mechanism by which PH complication accompanies GSD is unknown, we speculate two possible mechanisms. First, it has been reported that GSD-II is accompanied by glycogen accumulation in the smooth muscle cells of blood vessels and bladder, and vascular endothelial cells (Winkel et al., 2003; Thurberg et al., 2006). Since the *AGL* gene is expressed in these cell types (BioGPS, 2023), PVOD may develop because of the vascular occlusion caused by glycogen accumulation in the pulmonary vein. In addition, studies on bladder cancer and non-small cell lung cancer has shown that hyaluronic acid (HA) synthesis is enhanced in cancer tissues with decreased expression of *AGL* (Guin et al., 2016; Richmond et al., 2018). HA has a proliferative effect on endothelial cells and vascular smooth muscles (Kobayashi et al., 2020), and increased HA synthesis within smooth muscle cells of pulmonary arteries have been observed in patients with PH (Papakonstantinou et al., 2008). Since a decrease and/or loss in the AGL protein level in each cell type due to the *AGL* gene mutations is expected in the present case, endothelial and smooth muscle cell proliferation due to increased HA synthesis may be involved in the development of PVOD. Pathological evaluation of lung specimens at the time of lung transplantation in this case might help evaluate these hypotheses.

This study has some limitations. First, this case clearly meets the clinical diagnostic criteria for PVOD (Fukuda et al., 2019), but lacks a pathological diagnosis. Pulmonary vein obstruction may be confirmed using lung biopsy or pathological specimens, but thus far, the patient is still in the transplant waiting list, and lung biopsy could not be performed because of the risk of disease exacerbation. Second, the causal relationship between GSD-IIIb and PVOD has not yet been proven. Pathological examination or experimentation utilizing animal models possessing the same genetic mutations may assist in substantiating the correlation between the two diseases.

## Conclusion

We presented a case of GSD-IIIb complicated by PH due to PVOD and identified novel variants in the *AGL* gene. Although GSD-IIIb is generally considered to have a good prognosis, we should recognize the possibility of developing severe PH in later adulthood.

## Method (whole exome sequencing)

Genomic DNA was isolated from whole blood using the Qiagen QiaAmp Mini Prep Kit (Qiagen United States) according to manufacturer’s standard protocol. The whole-exome library was prepared using Sure Select All Exon V6 kit (Agilent, United States) according to manufacturer’s standard protocol. Sequencing was performed on Illumina Novaseq6000 platform (Illumina, United States) with 150 bp paired-end reads. Reads were trimmed using Trimmomatic v0.33 and aligned to reference genome hg38 by UCSC (original GRCh38 from NCBI, Dec. 2013) along with BWA v0.7.17. PCR duplicates were marked using Picard tools v2.18.2-SNAPSHOT. Variations were called using GATK tool (v4.0.5.1) and filtered variants were annotated using SnpEff (vSnpEff 4.3t 2017-11-24) and filtered with dbSNP(v151) and SNPs from the 1,000 genome project. In-house program and SnpEff are used to annotate with additional databases, including Exome Sequencing Progject (ESP6500SI_V2), ClinVar, dbNSFP (v3.5c), Exome Aggregation Consortium (ExAC), Genome Aggregation Database (gnomAD), 1,000 Genomes, and ACMG information. Mean depth of target regions was 122.4%x and 95.8% of the target regions had over 20x read depth. We filtered variants which are rare (MAF< 0.01 in the databases or not reported) and protein-altering. Two *AGL* null variants in this report (c.2308 + 2T>C and c.3045_3048dupTACC) were not previously reported in literature or databases we used in the analysis.

## Data Availability

The original contributions presented in the study are included in the article and Supplementary Material, further inquiries can be directed to the corresponding author.
