# Supplementary material for: Novel AGL variants in a patient with glycogen storage disease type IIIb and pulmonary hypertension caused by pulmonary veno-occlusive disease: A case report
Source: Front Genet. 2023 Mar 23;14:1148067. doi: 10.3389/fgene.2023.1148067 (PMC10078958; doi:10.3389/fgene.2023.1148067)

Supplementary Figure. Results of whole exome sequencing. Visualization of bam file using Integrative Genomics Viewer (IGV 2.16.0). (A) c.2308+2T>C (B) c.3045_3048dupTACC

A


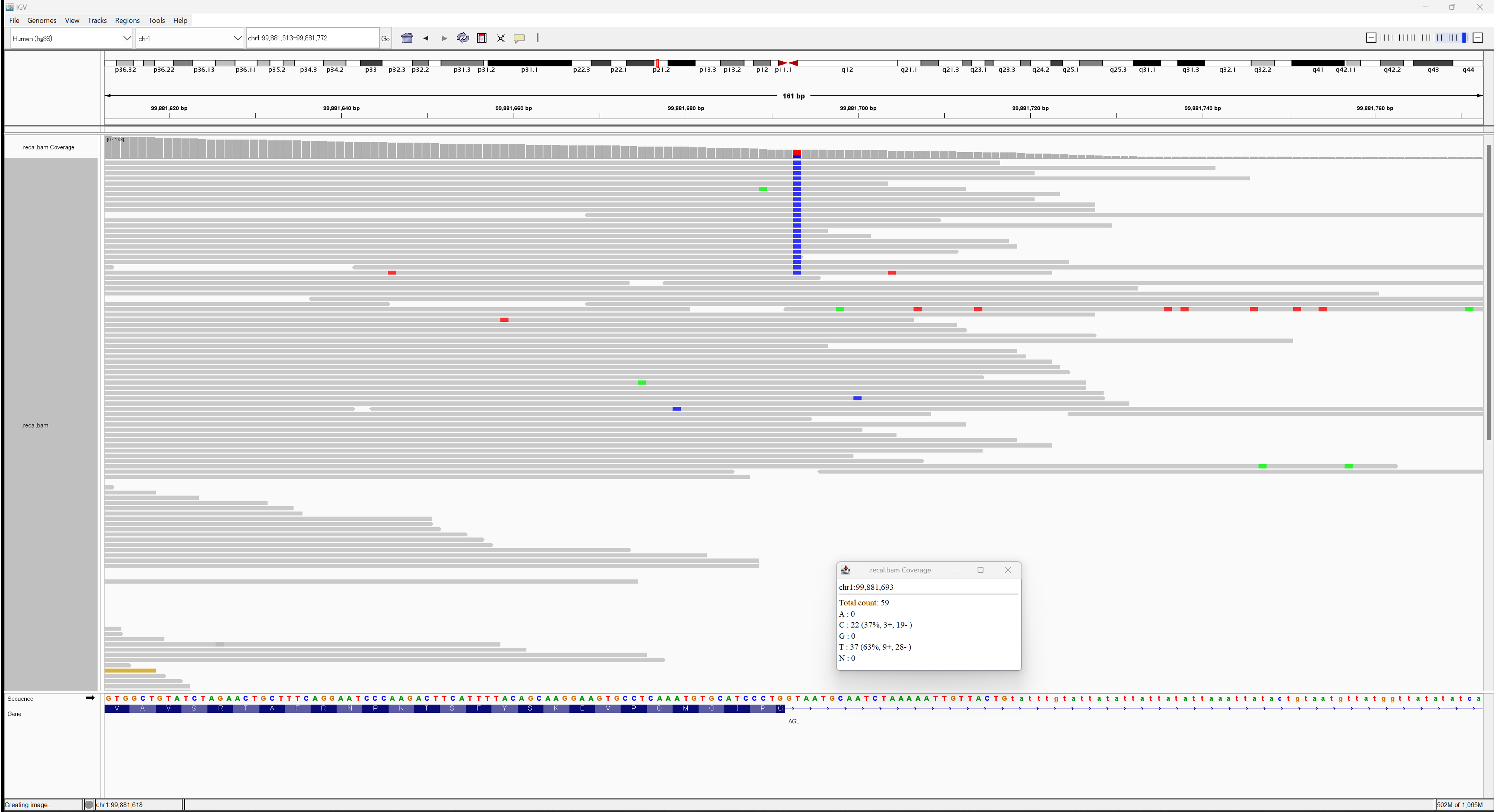


B


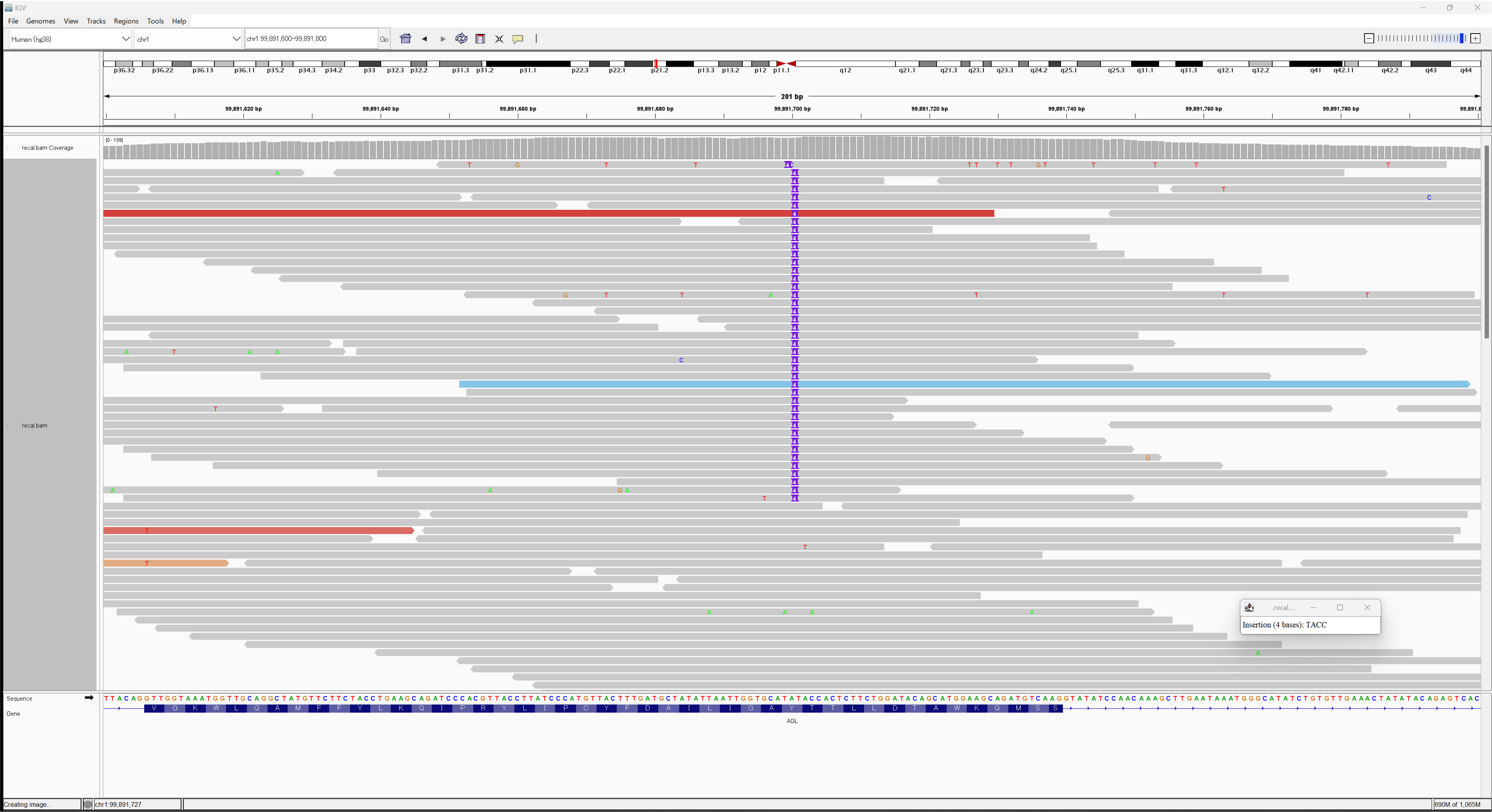

Supplement: Supplementary file 1 [file Table1.DOCX]
